# Supplementary material for: Adherent cells sustain membrane tension gradients independently of migration
Source: Nat Commun. 2025 Nov 26;16:10539. doi: 10.1038/s41467-025-65571-9 (PMC12657936; doi:10.1038/s41467-025-65571-9)
Supplement: Supplementary file 1 — Supplementary Information [file 41467_2025_65571_MOESM1_ESM.pdf]

Supplementary Figures

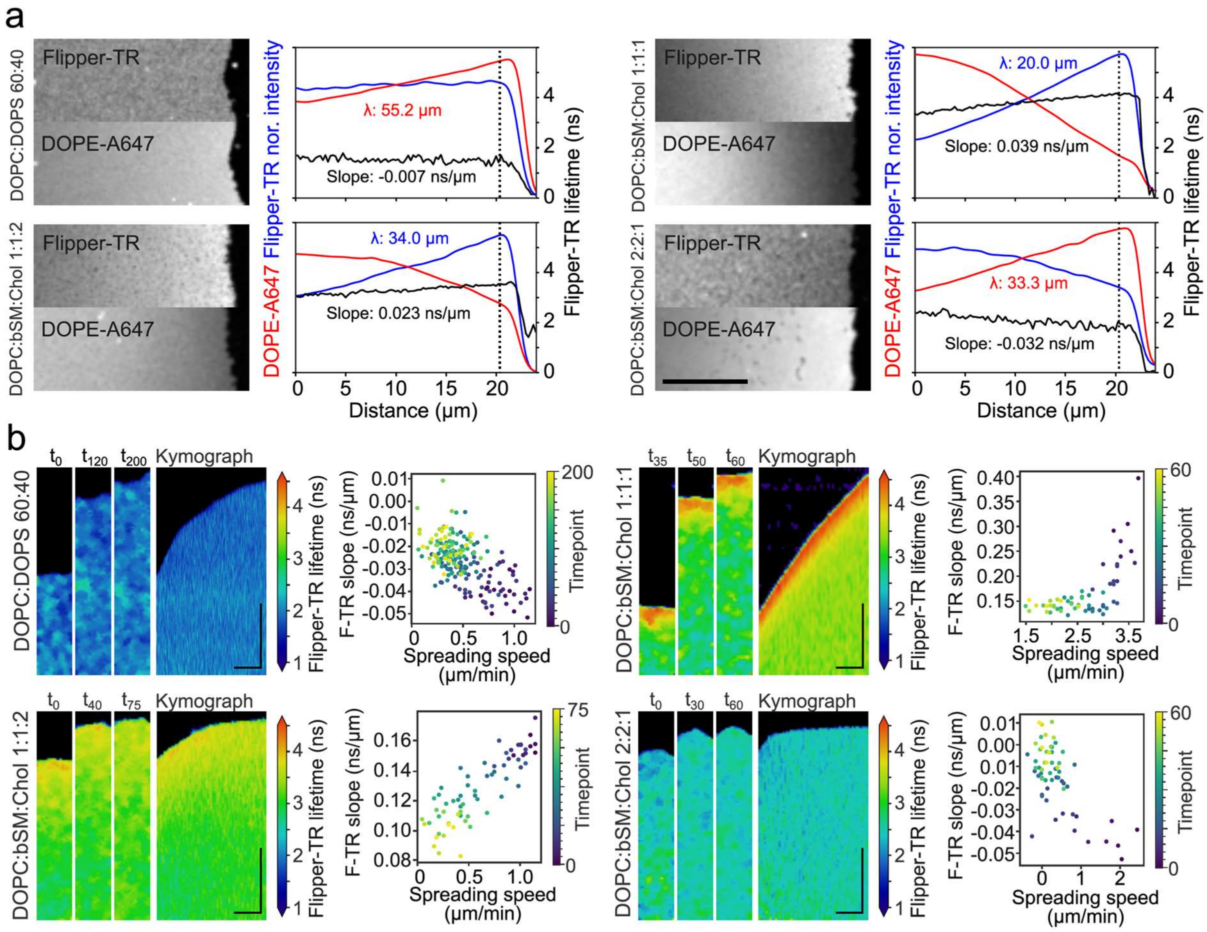

Supplementary Fig. 1: Flipper-TR reports membrane tension gradients in reconstituted membranes. A: Left, fluorescence confocal images of Flipper-TR and DOPE-atto647 in spreading bilayers of different compositions. Right, spatial profile of Flipper-TR (blue) and DOPE-atto647 (red) fluorescence intensity, and Flipper-TR average fluorescence lifetime (black, ns). Exponential (for fluorescence intensity, λ) or linear fits (for lifetime, slope) overlaid. Scale bar, 10 μm. **B**: Left, representative kymographs of expanding supported lipid bilayers of different compositions. Right, linear fits of spatial Flipper-TR gradients (ns/μm) over time Scale bar, 10 μm, 60s.

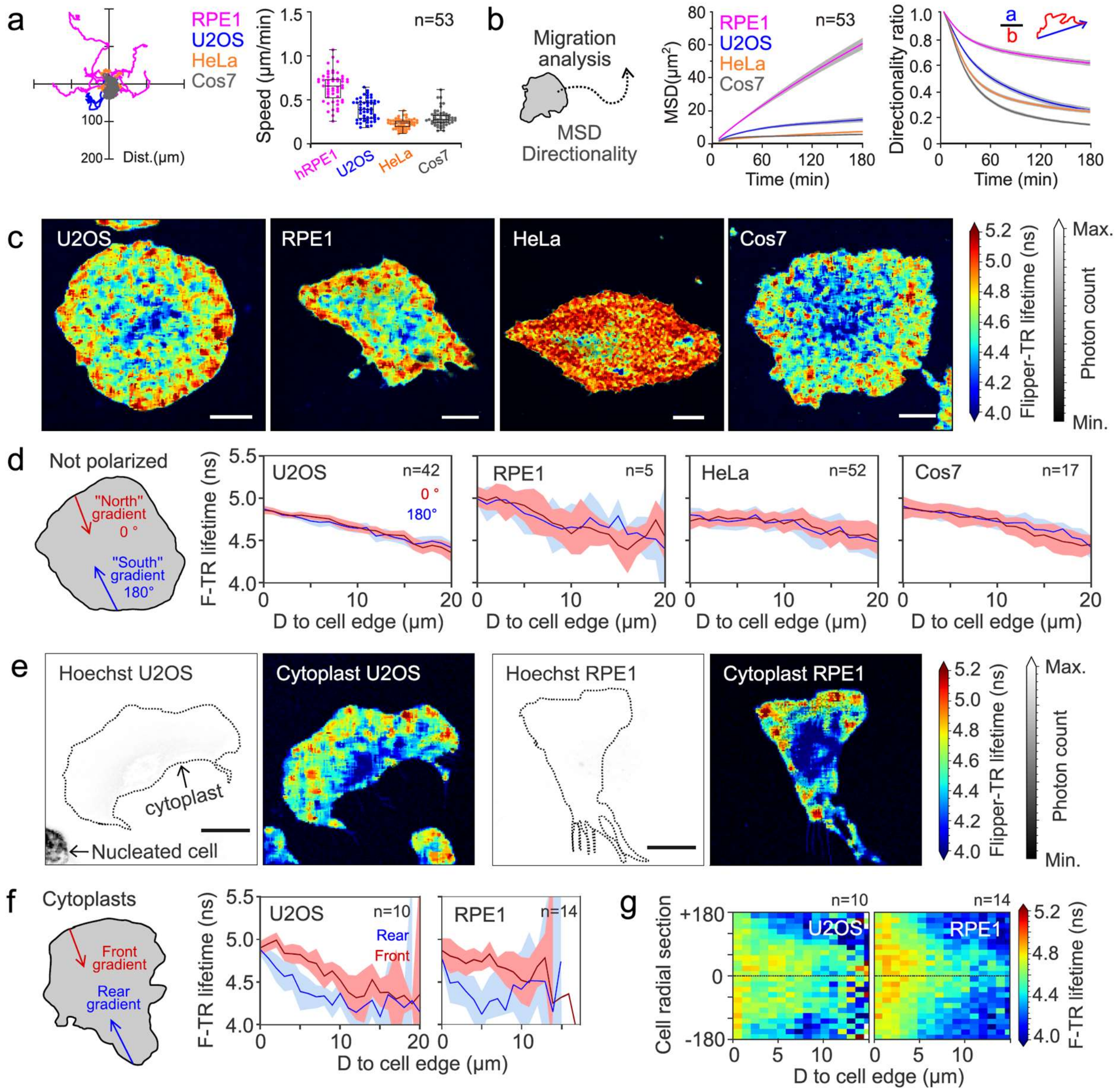

Supplementary Fig. 2: Leading-edge extension increases Flipper-TR lifetime in migrating cells. A: Mean square displacement (MSD, µm²) and directionality ratio as a function of elapsed time for RPE1 (magenta), U2OS (blue), HeLa (orange), and Cos7 (grey) cells, n=53. **B**: Instantaneous speed (µm/min) and trajectories over 180 minutes for RPE1 (magenta), U2OS (blue), HeLa (orange), and Cos7 (grey) cells, n=53. **C**: Representative image of non-polarized U2OS, RPE1, HeLa, and Cos7 cells labeled with Flipper-TR. **D**: Average Flipper-TR fluorescence lifetime as a function of D, distance from the edge at the front (red) and rear (blue) of non-polarized U2OS (n=90), RPE1 (n=37), HeLa (n=11), and Cos7 (n=15) cells. Line represents mean ± standard deviation. **E**: Representative image of U2OS and RPE1 cytoplasts labeled with Flipper-TR and Hoechst. **F**: Average Flipper-TR fluorescence lifetime as a function of D, distance from the edge at the front (red) and rear (blue) of U2OS (n=10) and RPE1 (n=14) cytoplasts. Line represents mean ± standard deviation. **G**: Average Flipper-TR fluorescence lifetime as a function of D, distance from the edge and radial position (front at 0°) of U2OS (n=10) and RPE1 (n=14) cytoplasts. Color indicates average Flipper-TR fluorescence lifetime (ns). **A,C,E**: Scale bar, 10 µm.

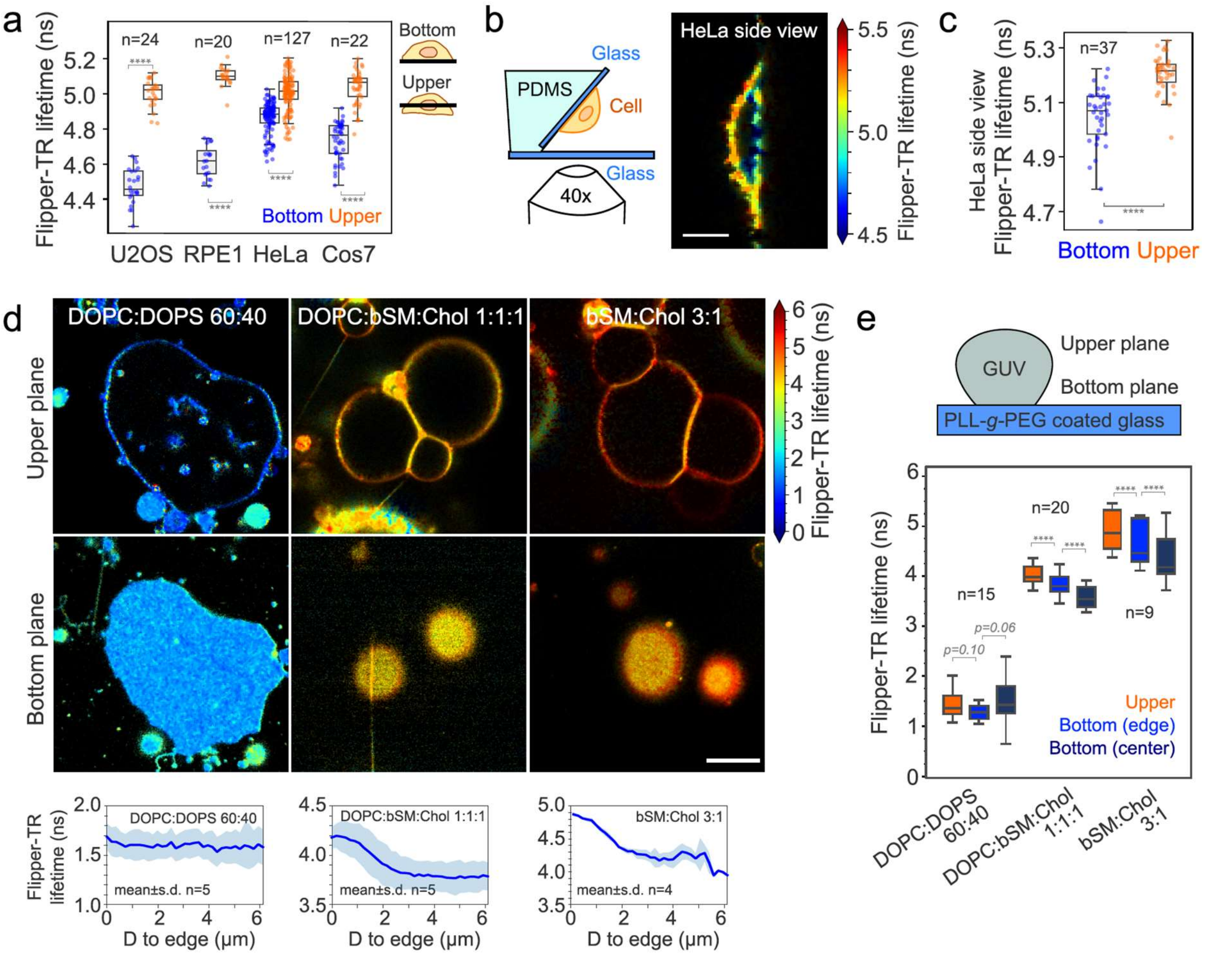

Supplementary Fig. 3: Apicobasal tension gradients *in vitro* and in cells. A: Average Flipper-TR fluorescence lifetime (ns) at bottom and upper (+3 µm) planes in different cell types. Grey, Welch's P value ($p<10^{-4}$). N=3. **B:** Schematic of the experimental set-up of the side view experiments. Cells are seeded on a fibronectin-coated glass coverslip help oblique to the water-immersion objective. **C:** Average Flipper-TR fluorescence lifetime (ns) at bottom and upper regions of HeLa cells in side view. Grey, Welch's P value ($p<10^{-4}$). **D:** Top, representative confocal FLIM image of GUVs labeled with Flipper-TR, adhered on PLL-*g*-PEG-coated glass, on upper and bottom planes. Color indicates average Flipper-TR fluorescence lifetime (ns). Bottom, average Flipper-TR fluorescence lifetime (ns) as a function of D, distance from the edge at the bottom plane of adhered GUVs with different compositions. **E:** Top, schematic of the experimental set-up of adhered GUVs. Bottom, average Flipper-TR fluorescence lifetime (ns) at the upper and edges and center of bottom plane of adhered GUVs with different compositions. **B,D**: Scale bar, 10 µm.

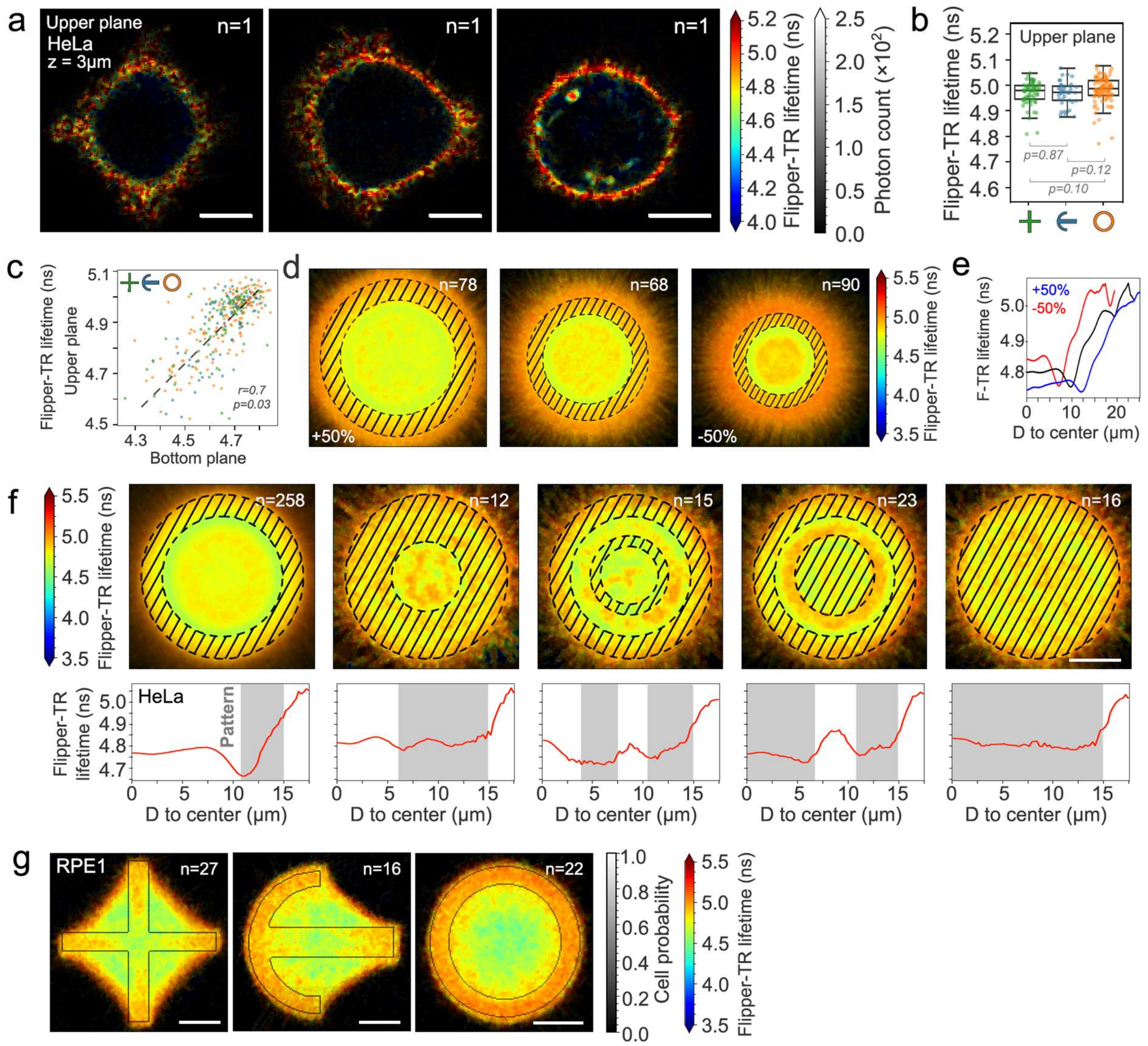

Supplementary Fig. 4: Membrane tension gradients are shaped by cellular organization. **A:** Representative confocal FLIM images at the upper plane (3 μm from glass surface) of HeLa cells labeled with Flipper-TR on cross, crossbow, and ring micropatterns. **B:** Average Flipper-TR fluorescence lifetime at the upper plane of cross, crossbow, and ring micropatterned cells (n=130, 52, 161, Welch's P). Data distribution in black. **C:** Average Flipper-TR fluorescence lifetime (ns) at bottom plane vs. upper plane of cross (green), crossbow (blue), and ring (orange) micropatterned cells. Labels refer to r and p values from linear fit. **D:** Cell averaging of average Flipper-TR lifetime in bottom membrane HeLa cells on ring micropatterns of different sizes. Stripped areas represent adhesive micropattern location**.** **E:** Average Flipper-TR lifetime as a function of the distance from the center of ring micropatterns of different sizes. **F** Top, cell averaging of average Flipper-TR lifetime in bottom membrane of patterned HeLa cells on different motifs. Bottom, average Flipper-TR lifetime vs. distance from pattern center. Shaded areas represent adhesive micropattern location. **G:** Average confocal FLIM images at the bottom plane of RPE1 cells labeled with Flipper-TR on cross, crossbow, and ring micropatterns.

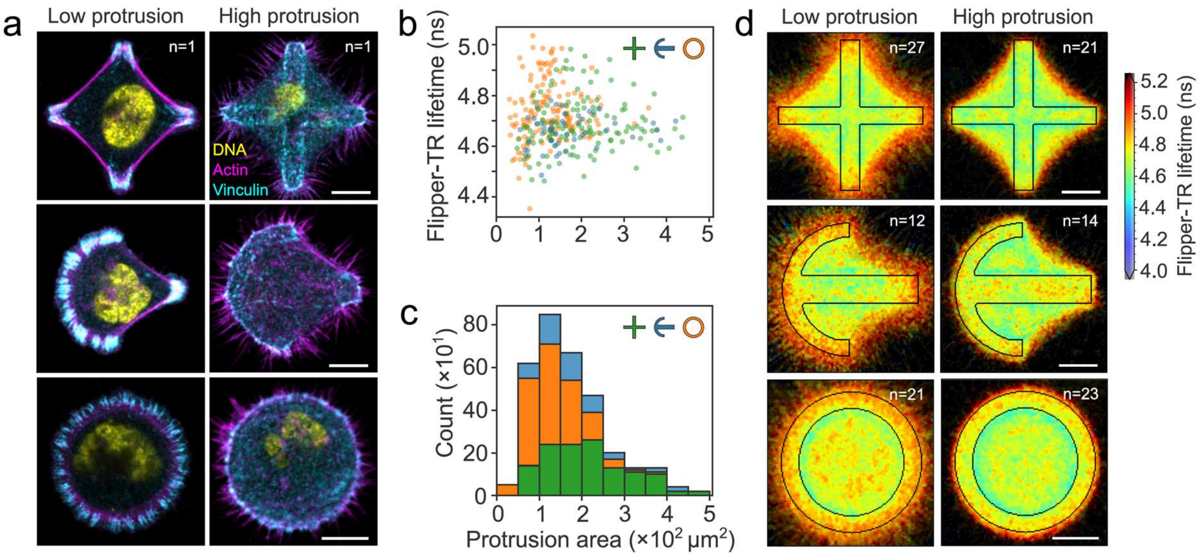

Supplementary Fig. 5: Protrusion extension increases membrane tension. A: Representative fluorescence images at basal plane of cross, crossbow, and ring micropatterned cells stained with phalloidin (magenta), vinculin antibody (cyan), and Hoechst (yellow) showing low (left column) or high number of protrusions (right). **B**: Average Flipper-TR lifetime (ns) per cell at bottom membrane vs. protrusion area (μm²) of cross (green), crossbow (blue), and ring (orange) micropatterned HeLa cells. **C**: Histogram of protrusion areas (μm²) of cross (green), crossbow (blue), and ring (orange) micropatterned HeLa cells. **D**: Average Flipper-TR lifetime map in the bottom membrane of micropatterned HeLa cells with high (top) or low (bottom) number of protrusions. **A,D**: Scale bar, 10 μm.

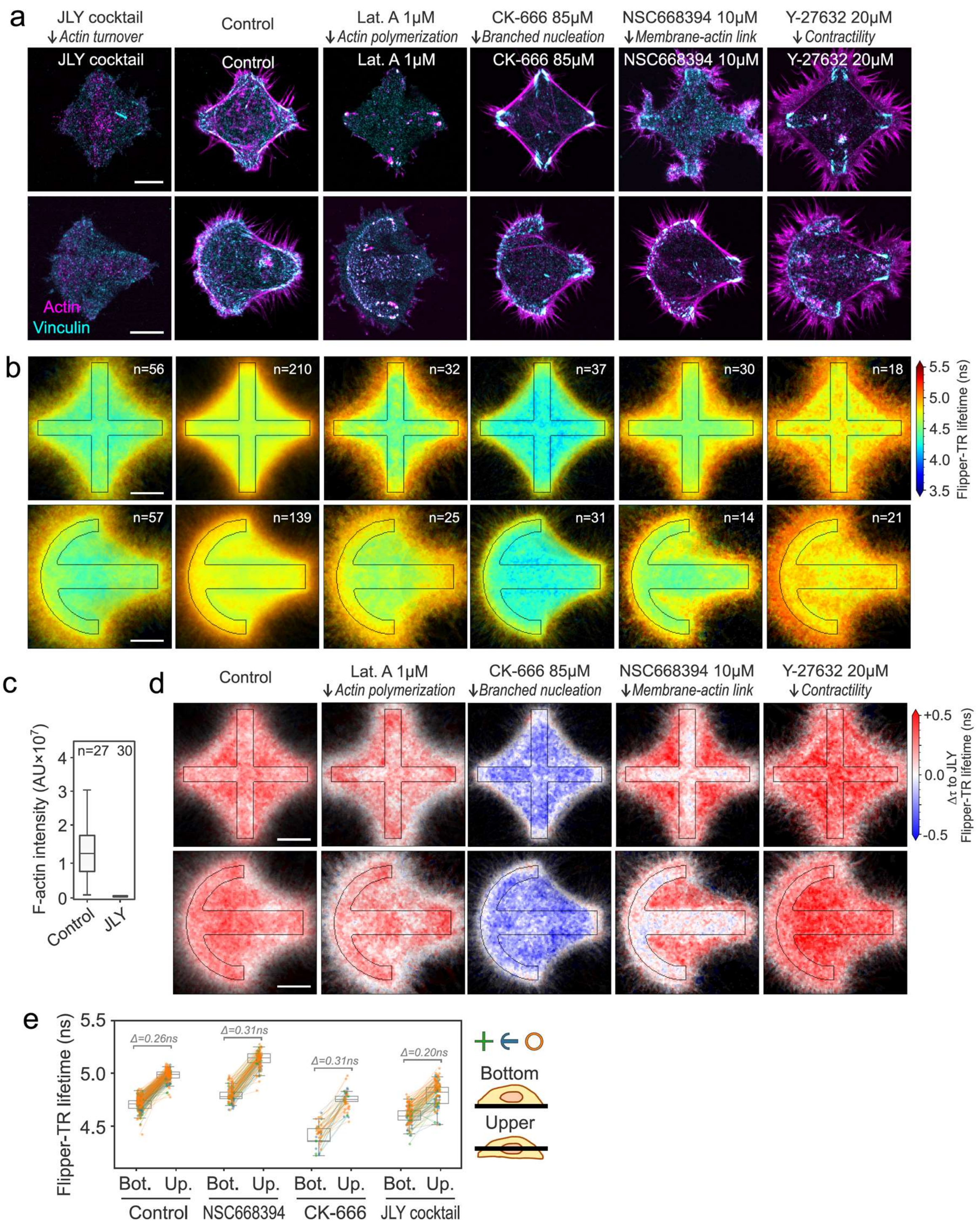

Supplementary Fig. 6: A: Representative fluorescence images at bottom plane of HeLa cells on cross and crossbow micropatterns stained with phalloidin (magenta) and vinculin antibody (cyan) under different drug treatments. **B:** Average confocal FLIM images at the bottom plane of HeLa cells labeled with Flipper-TR on cross and crossbow micropatterns under different drug treatments. **C:** Integrated intensity of phalloidin staining (F-actin) in control and JLY-treated micropatterned cells. **D:** Flipper-TR lifetime difference respective to JLY treatment. **E:** Average Flipper-TR lifetime (ns) at bottom/upper planes of cross (green), crossbow (blue), and ring (orange) micropatterned HeLa cells under different drug treatments. Labels display difference from control in ns. Lines represent data pairing. Data distribution in black.

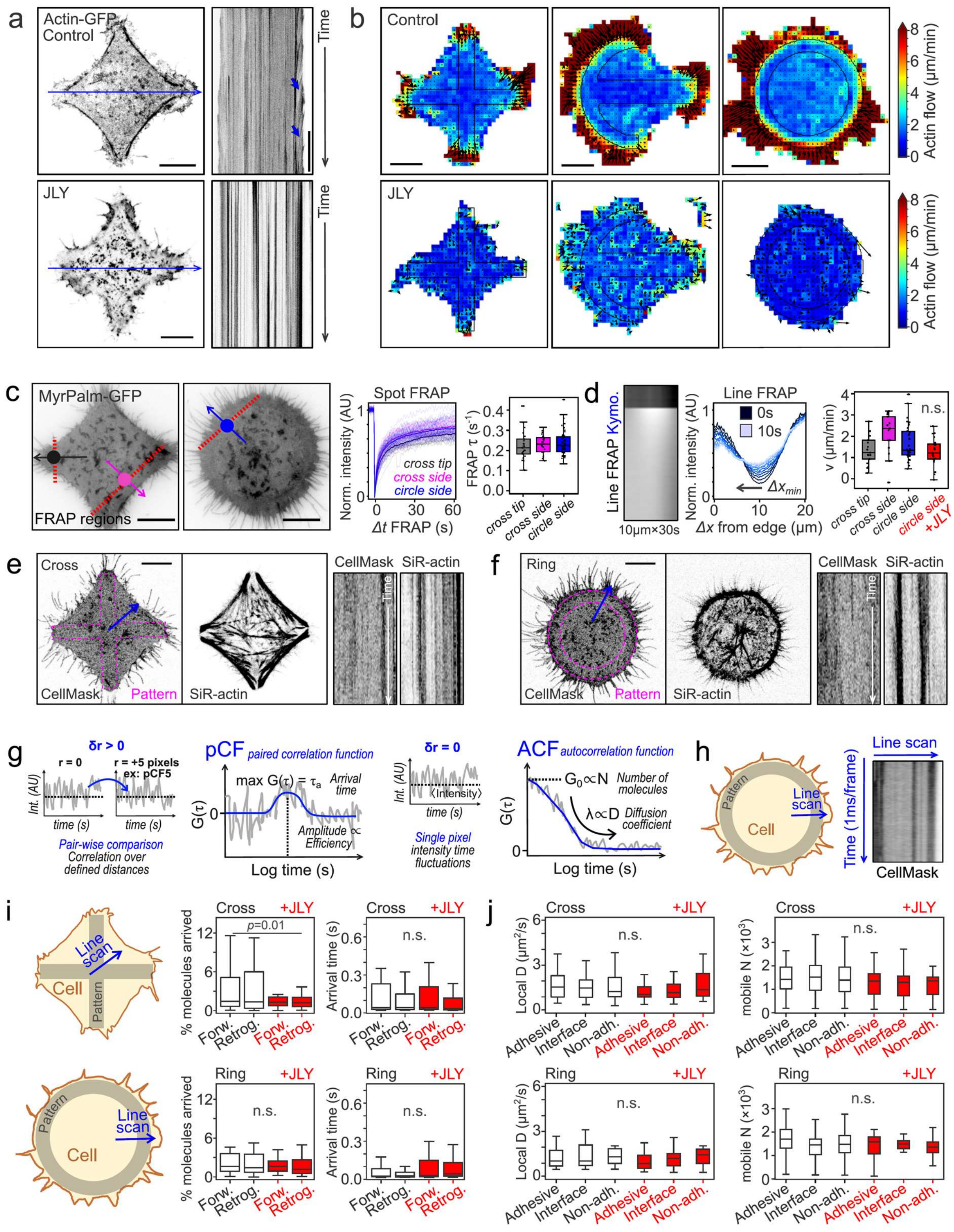

Supplementary Fig. 7: Lipid diffusion and flows are limited in patterned cells. A: Left, representative images at basal plane of cross micropatterned actin-GFP HeLa cells under control (top) and JLY (bottom) conditions. Blue line indicates kymograph position. Blue arrows mark retrograde flow events. Scale bar, 1 min. **B**: Cell averaging of particle image velocimetry analysis of actin-GFP HeLa cells on cross, crossbow, and ring micropatterns under control (top, n=10,10,12 respectively) and JLY treatments (bottom n=4,4,4 respectively). Color and arrow lengths indicate cortical flow magnitude. **C:** Left, FRAP

experimental design overlaid on images of HeLa MyrPalm-GFP on cross and ring micropatterns. Line FRAP bleaching sites marked with red dotted lines; recovery tracked along black, magenta, and blue arrows. Middle, normalized fluorescence intensity over time after FRAP for spots at cross tip (grey), cross side (magenta), or ring side (blue). Shaded lines represent individual time traces. Right, FRAP recovery constant (s^{-1}). **D**: Line FRAP kymograph (inverted LUT) of ring cell from panel A and intensity vs. distance from edge, colored by time elapsed from FRAP. Right, velocity (µm/min) of fluorescence minima vs. distance to cell edge. Statistical test: Welch's *P* value=0.04. **E-F**: Cell mask and SiR-actin images of HeLa cells on cross (panel C) or ring (panel D) pattern. Kymographs from blue arrows are shown on the right. **G:** Parameters calculated from pair-correlation function (pCF, left) and autocorrelation function fits (ACF, right). **H:** Schematics of the acquisition of line scans, and representative line scan from a cell mask-stained HeLa cell. **I-J**: Schematics of line scan acquisition and fluorescence correlation analysis of patterned HeLa cells stained with CellMask dye under control (black) and JLY (red) conditions. Mean values ± standard deviation. **I**: pCF analysis of cross (top) or ring (bottom) patterned cells. First column, percentage of molecules arrived. Second column, arrival time (s). **J**: ACF analysis of cross (top) or ring (bottom) patterned cells. First column, diffusion coefficient ($\mu m^2/s$). Second column, number of moving molecules (in thousands). **A-C,E,F**: Scale bar, 10 µm.

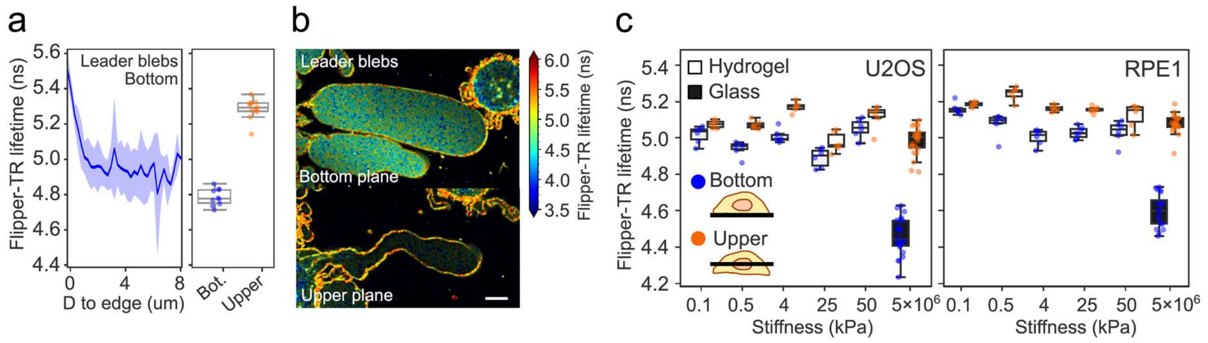

Supplementary Fig. 8: A: Average Flipper-TR fluorescence lifetime (ns) as a function of D, distance from the edge at the bottom plane of stable blebs from HeLa cells confined to 3 µm under non-adhesive conditions. **B:** Representative confocal FLIM images of HeLa cells confined to 3 µm under non-adhesive conditions (note the tilt on the bottom-plane image due to chamber compression). Scale bar, 10 µm. **C:** Average Flipper-TR lifetime (ns) at the top and bottom planes of U2OS and RPE1 cells stained with Flipper-TR and a function of substrate stiffness (kPa). Median, quartile distribution, and individual data points are shown. Statistical test: Welch's p value.
