# Supplementary material for: Adherent cells sustain membrane tension gradients independently of migration
Source: Nat Commun. 2025 Nov 26;16:10539. doi: 10.1038/s41467-025-65571-9 (PMC12657936; doi:10.1038/s41467-025-65571-9)
Supplement: Supplementary file 2 — Reporting Summary [file 41467_2025_65571_MOESM2_ESM.pdf]

nature portfolio

Corresponding author(s): Garcia-Arcos, Roux

Last updated by author(s): Sep 22, 2025

Reporting Summary

Nature Portfolio wishes to improve the reproducibility of the work that we publish. This form provides structure for consistency and transparency in reporting. For further information on Nature Portfolio policies, see our Editorial Policies and the Editorial Policy Checklist.

Statistics

For all statistical analyses, confirm that the following items are present in the figure legend, table legend, main text, or Methods section.

n/a	Confirmed	
☐	☒	The exact sample size (*n*) for each experimental group/condition, given as a discrete number and unit of measurement
☐	☒	A statement on whether measurements were taken from distinct samples or whether the same sample was measured repeatedly
☐	☒	The statistical test(s) used AND whether they are one- or two-sided<br>*Only common tests should be described solely by name; describe more complex techniques in the Methods section.*
☒	☐	A description of all covariates tested
☐	☒	A description of any assumptions or corrections, such as tests of normality and adjustment for multiple comparisons
☐	☒	A full description of the statistical parameters including central tendency (e.g. means) or other basic estimates (e.g. regression coefficient) AND variation (e.g. standard deviation) or associated estimates of uncertainty (e.g. confidence intervals)
☐	☒	For null hypothesis testing, the test statistic (e.g. *F*, *t*, *r*) with confidence intervals, effect sizes, degrees of freedom and *P* value noted<br>*Give P values as exact values whenever suitable.*
☒	☐	For Bayesian analysis, information on the choice of priors and Markov chain Monte Carlo settings
☒	☐	For hierarchical and complex designs, identification of the appropriate level for tests and full reporting of outcomes
☒	☐	Estimates of effect sizes (e.g. Cohen's *d*, Pearson's *r*), indicating how they were calculated

Our web collection on statistics for biologists contains articles on many of the points above.

Software and code

Policy information about availability of computer code

Data collection	For confocal fluorescence lifetime imaging we used Symphotime (PicoQuant) integrated on Nikon Elements software. Standard scanning confcal was acquired on NIS. Azimutal TIRF was acquired usng the VisiView software (Visitron Systems). Mass spectrometry was recorded using in the Tune software (Thermo Fisher Scientific)
Data analysis	Basic image analysis and format conversion was performed on ImageJ/Fiji software. Images were imported from NIS Elements (.nd2 format) using the BioFormats plugin or exported as ome.tiff format. Custom Python scripts were used for data analysis as Jupyter notebooks, and available on the online repository along with all input/output data needed to replicate the plots.

For manuscripts utilizing custom algorithms or software that are central to the research but not yet described in published literature, software must be made available to editors and reviewers. We strongly encourage code deposition in a community repository (e.g. GitHub). See the Nature Portfolio guidelines for submitting code & software for further information.

Data

Policy information about availability of data

All manuscripts must include a data availability statement. This statement should provide the following information, where applicable:

- Accession codes, unique identifiers, or web links for publicly available datasets
- A description of any restrictions on data availability
- For clinical datasets or third party data, please ensure that the statement adheres to our policy

All original microscopy images and datasets shown in the figures, and all original code and associated datasets have been deposited at an open repository and will

be made publicly available as of the date of publication. Any additional data related to this paper will be shared by the lead contact upon request.
Figshare data DOI: https://doi.org/10.6084/m9.figshare.26304718
Any additional information required to reanalyze the data reported in this paper is available from the lead contact upon request.

Research involving human participants, their data, or biological material

Policy information about studies with human participants or human data. See also policy information about sex, gender (identity/presentation), and sexual orientation and race, ethnicity and racism.

Reporting on sex and gender	No human data
Reporting on race, ethnicity, or other socially relevant groupings	No human data
Population characteristics	No human data
Recruitment	No human data
Ethics oversight	No human data

Note that full information on the approval of the study protocol must also be provided in the manuscript.

Field-specific reporting

Please select the one below that is the best fit for your research. If you are not sure, read the appropriate sections before making your selection.

- [x] Life sciences
- [ ] Behavioural & social sciences
- [ ] Ecological, evolutionary & environmental sciences

For a reference copy of the document with all sections, see nature.com/documents/nr-reporting-summary-flat.pdf

Life sciences study design

All studies must disclose on these points even when the disclosure is negative.

Sample size	No sample size calculation was performed. Sample size is not a limiting factor in our study; we took a high number of cells/mesurements to represent the spread of data.
Data exclusions	No exclusion of data was applied.
Replication	We performed N>3 replicates and indicated in each panel, replicates were never excluded from data pooling.
Randomization	We randomly selected cells in drug-treatment/control conditions or all cells in the dish/patterns
Blinding	Blinding was performed only in some datasets as for the supported lipid bilayer data or the FLIM of micropatterned cells. Blinding was performed in one of the replicates to ensure there was no experimenter's bias.

Reporting for specific materials, systems and methods

We require information from authors about some types of materials, experimental systems and methods used in many studies. Here, indicate whether each material, system or method listed is relevant to your study. If you are not sure if a list item applies to your research, read the appropriate section before selecting a response.

Materials & experimental systems

n/a	Involved in the study	
[ ]	[x]	Antibodies
[ ]	[x]	Eukaryotic cell lines
[x]	[ ]	Palaeontology and archaeology
[ ]	[x]	Animals and other organisms
[x]	[ ]	Clinical data
[x]	[ ]	Dual use research of concern
[x]	[ ]	Plants

Methods

n/a	Involved in the study	
[x]	[ ]	ChIP-seq
[x]	[ ]	Flow cytometry
[x]	[ ]	MRI-based neuroimaging

Antibodies

Antibodies used	Anti-Vinculin Vinculin Monoclonal Antibody (7F9)-Alexa Fluor488 at 1:25 dillution (eBioscience Thermofisher #53-9777-82)

Validation	Manufacturer's wesite states: vinculin Antibody (7F9) is recommended for detection of vinculin of human, mouse, rat and avian origin by WB, IP, IF and IHC(P). The antibody has been cited over 500 times. Fro example at PMID: 28831175, Figure 2.

Eukaryotic cell lines

Policy information about cell lines and Sex and Gender in Research

Cell line source(s)	Human cervical adenocarcinoma cells HeLa-Kyoto stably expressing myosin IIA (Myh9)-GFP and LifeAct-mCherry or Myh9-GFP, or LifeAct-mCherry and the plasma membrane-targeting CAAX box fused to GFP were a gift from the lab of Matthieu Piel (Institut Curie, Paris, France). TALEN-edited ActB fused with GFP were sourced form the lab of Matthieu Piel, originally from the company Cellectis, Paris, France.<br>HeLa (human female), RPE1 (human female), Cos7 (male monkey) and U2OS cells (human female) for migration studies with no stable marker were sourced from ATCC in the lab of Charlotte Aumeier.
Authentication	HeLa, RPE1, Cos7 and U2OS cells for migration studies with no stable marker were sourced from ATCC. Other cell lines were not authenticated.
Mycoplasma contamination	All cell lines were tested for mycoplasma contamination using Mycoplasmacheck PCR Detection (Eurofins, #50400400), and treated once a year with Plasmocin profilactic (InvivoGen, #ant-mpp) and treatment Mycoplasma Elimination Reagents (InvivoGen, #ant-mpt).
Commonly misidentified lines (See ICLAC register)	No commonly misidentified lines we used in this study.

Animals and other research organisms

Policy information about studies involving animals; ARRIVE guidelines recommended for reporting animal research, and Sex and Gender in Research

Laboratory animals	Keratocyte cells were extracted from scales of zebrafish (AB adults Danio Rerio) of male and female individuals.
Wild animals	*Provide details on animals observed in or captured in the field; report species and age where possible. Describe how animals were caught and transported and what happened to captive animals after the study (if killed, explain why and describe method; if released, say where and when) OR state that the study did not involve wild animals.*
Reporting on sex	*Indicate if findings apply to only one sex; describe whether sex was considered in study design, methods used for assigning sex. Provide data disaggregated for sex where this information has been collected in the source data as appropriate; provide overall numbers in this Reporting Summary. Please state if this information has not been collected. Report sex-based analyses where performed, justify reasons for lack of sex-based analysis.*
Field-collected samples	*For laboratory work with field-collected samples, describe all relevant parameters such as housing, maintenance, temperature, photoperiod and end-of-experiment protocol OR state that the study did not involve samples collected from the field.*
Ethics oversight	This protocol was carried out by trained personnel at the Zebrafish Core Facility of the medical center of the University of Geneva. Ethical approval was not required for this study because no animals were subjected to additional experimental procedures. Zebrafish scales were obtained exclusively as residual material from animals already used in ethically approved studies conducted at the University of Geneva under approval of CARE committee.

Note that full information on the approval of the study protocol must also be provided in the manuscript.

Plants

Seed stocks	N/A
Novel plant genotypes	N/A
Authentication	N/A
