# Supplementary material for: Adherent cells sustain membrane tension gradients independently of migration
Source: Nat Commun. 2025 Nov 26;16:10539. doi: 10.1038/s41467-025-65571-9 (PMC12657936; doi:10.1038/s41467-025-65571-9)
Supplement: Supplementary file 3 — Description of Additional Supplementary Files [file 41467_2025_65571_MOESM3_ESM.pdf]

Description of Additional Supplementary files

Supplementary Video 1. Expanding supported lipid bilayers (SLBs) with different compositions.

Supplementary Video 2. Confocal FLIM time lapses of migrating U2OS cells stained with Flipper-TR.

Supplementary Video 3. Actin dynamics in patterned HeLa Actin-GFP cells, examples of lamellipodium and stress fiber dominated phenotypes.

Supplementary Video 4. Actin dynamics in patterned HeLa Actin-GFP cells in control(+DMSO) versus JLY-treated conditions.
